# Study of Hybrid PVA/MA/TEOS Pervaporation Membrane and Evaluation of Energy Requirement for Desalination by Pervaporation

**DOI:** 10.3390/ijerph15091913

**Published:** 2018-09-03

**Authors:** Zongli Xie, Derrick Ng, Manh Hoang, Jianhua Zhang, Stephen Gray

**Affiliations:** 1 CSIRO Manufacturing, Private Bag 10, Clayton South, VIC 3169, Australia; derrick.ng@csiro.au (D.N.); manh.hoang@csiro.au (M.H.); 2 Institute for Sustainability and Innovation, Victoria University, PO Box 14428, Melbourne, VIC 8001, Australia; jianhua.zhang@vu.edu.au (J.Z.); stephen.gray@vu.edu.au (S.G.)

**Keywords:** pervaporation, desalination, energy, hybrid organic-inorganic membrane, PVA/MA/silica

## Abstract

Desalination by pervaporation is a membrane process that is yet to be realized for commercial application. To investigate the feasibility and viability of scaling up, a process engineering model was developed to evaluate the energy requirement based on the experimental study of a hybrid polyvinyl alcohol/maleic acid/tetraethyl orthosilicate (PVA/MA/TEOS) Pervaporation Membrane. The energy consumption includes the external heating and cooling required for the feed and permeate streams, as well as the electrical power associated with pumps for re-circulating feed and maintaining vacuum. The thermal energy requirement is significant (e.g., up to 2609 MJ/m^3^ of thermal energy) and is required to maintain the feed stream at 65 °C in recirculation mode. The electrical energy requirement is very small (<0.2 kWh/m^3^ of required at 65 °C feed temperature at steady state) with the vacuum pump contributing to the majority of the electrical energy. The energy required for the pervaporation process was also compared to other desalination processes such as Reverse Osmosis (RO), Multi-stage Flash (MSF), and Multiple Effect Distillation (MED). The electrical energy requirement for pervaporation is the lowest among these desalination technologies. However, the thermal energy needed for pervaporation is significant. Pervaporation may be attractive when the process is integrated with waste heat and heat recovery option and used in niche applications such as RO brine concentration or salt recovery.

## 1. Introduction

Increasing population and global warming have created greater disparities between the supplies and demands of fresh water sources. Seawater and brackish water desalination technologies have been used to overcome water scarcity issues by providing reliable fresh water [1]. Major desalination technologies include Reverse Osmosis (RO), Electrodialysis Reversal (EDR), Multi-stage Flash (MSF), Multiple Effect Distillation (MED), and Vapor Compression (VC). Each has its advantages and disadvantages and the choice of which technology to use is highly dependent on the requirement at hand, the use of different energy sources and restrictions faced at the specific site. Karagiannis and Soldatos [2] conducted an extensive literature review on water desalination cost for different desalination technologies. Cost estimates seem to be very much site specific and the water production cost ranges from installation to installation because the water cost depends upon many factors including the desalination method, the level of feed water salinity, the energy source, and the capacity of the desalination plant. Thermal methods such as MSF and MED are generally adopted in Gulf Countries and only financially viable in larger scale seawater desalination plants with high capital cost [2]. Their energy consumption is generally high regardless of the level of salt concentration and it is therefore not a viable option for brackish water desalination [3]. VC is used mainly for small systems with production around 1000 m^3^/day [2]. For EDR, the major energy requirement is the direct current used to separate the ionic substances in the membranes stack and approximately 1 kWh electrical energy is required to extract 1 kg of salt [3]. Because the power consumption of EDR is directly proportional to the feed water salinity, it is mostly suitable for brackish feed waters. In the last two decades, with advances in membrane materials and improvement in energy recovery, RO technology has improved considerably and more RO plants are being constructed throughout the world [3]. RO accounts for >65% of total world desalination capacity and distillation (mainly MSF) accounts for about 30% [4].

Table 1 compares these typical desalination technologies including typical capital and operating costs. In general, the capital costs for RO plants tend to be lower than for MED and EDR plant of similar capacity. Thermal technologies are used mainly for large capacity (>300–400 kL/day) plants with very high feed water TDS (>30,000 mg/L) [2]. On the other hand, membrane methods, mainly RO, are used by medium and low capacity systems. RO is dominant in the desalination of brackish water due to its low energy consumption and consequently its low cost advantage [2]. EDR systems are only economically viable over RO when the feed water TDS is between 3000 to 12,000 mg/L, the plant capacity required is greater than 100 kL/d, and the feed water is highly scaling [3]. At present, RO is the most energy-efficient technology for seawater desalination and is the benchmark for comparison for any new desalination technology [5].

Fiorenza et al. [6] claimed that the typical average capacity and corresponding costs for seawater desalination technologies in the world was:

- MSF: 25,000 m^3^/day and $1.10/m^3^
- MED: 10,000 m^3^/day and $0.80/m^3^
- VC: 3000 m^3^/day and $0.70/m^3^
- RO: 6000 m^3^/day and $0.70/m^3^

The energy requirement for seawater RO operation is significant, attributing to as much as 23% of total water cost [3]. The osmotic pressure for seawater of salinity 35,000 mg/L is 2800 kPa, whereas the osmotic pressure is 140 kPa for brackish water of salinity 1600 mg/L [4]. This means that for seawater RO, a significantly higher pressure must be applied to prevent osmotic transfer of water through the semi-permeable membrane, and consequently a high energy consumption results. In addition, the water recovery for RO is generally low (less than 50% for seawater RO). Several technologies including forward osmosis (FO), membrane distillation (MD), capacitive deionization (CDI) and pervaporation (PV) have been proposed as potential alternative desalination processes in recent years [5]. These processes have not yet achieved extensive commercial success and viability as they still need to compete with RO and MED. However, under certain circumstances, they could prove to be viable due to their unique advantages. For example, FO relies on osmosis, the natural diffusion of water through a semipermeable membrane from a low concentration solution to a draw solution having a higher concentration of dissolved material. Driven by an osmotic pressure gradient, FO does not require significant energy input as it operates at low or no hydraulic pressure [7]. The challenges for FO mainly focus on developing new draw solutions that are of high osmotic pressure, yet are easily recoverable. CDI is a relatively new as a low energy process for brackish freed waters, which is an electro-adsorption process aimed at low salinity waters. It claims high water recovery (75–85%) at low energy cost (less than a third of RO) [8]. However, its capital cost remains high and it is only viable for low salinity water desalination. This method may be suitable for small-scale use in remote regions, because minimal pretreatment of the feed is needed, but the energy consumption for large-scale sea water desalination will likely exceed RO [5]. MD is a thermally driven process involving the transport of water vapor through a hydrophobic membrane and rejects the liquid phase. MD offers the attractiveness of operation at atmosphere pressure and low temperatures (30–80 °C), with the theoretical ability to achieve 100% salt rejection [9]. An allied process to MD, PV involves a dense hydrophilic membrane rather than porous membrane, and the driving force is the vapor pressure difference between the feed solution and the permeate vapor, which is normally maintained by applying a vacuum on the permeate side. Similar to MD, it also offers advantages of high salt rejection and energy needs practically independent of the feed salinity [10]. This implies that PV could be potentially used in applications where RO has limitations, such as brine concentrate management or for zero liquid waste technology.

The performance of PV, like other membrane processes, mainly depends on: (1) the membrane properties, (2) the operating conditions, and (3) the module design [11]. There are extensive studies available that focus on the effect of membrane properties and operating conditions. However, only few studies refer to the energy required and economics of the pervaporation process [7,8]. Researchers such as Servel et al. [12] have calculated energy required by conventional distillation column and hybrid column-PV system for the dehydration of acetic acid via software like Aspen, they concluded that 20% of energy gain was possible in principle using the hybrid system. The economy of the pervaporation process can be assessed with following parameters: the specific energy required for heating, the specific power required for circulating feed and vacuum pump operation, and the specific membrane area [9]. The energy used for desalination by pervaporation is primarily heat and electricity. For good performance of PV, high water flux must be obtained with moderate energy consumption as lower water flux requires a higher installed membrane area and consequently increases the membrane associated capital cost.

This study aims to study the performance of a hybrid polyvinyl alcohol/maleic acid/tetraethyl orthosilicate (PVA/MA/TEOs) membrane for desalination and evaluate the energy consumption required for desalination by PV. The energy consumption considered in this work refers to the external heating/cooling required for feed/permeate stream, as well as the energy consumption associated with pumps for re-circulating feed and maintaining vacuum. The energy required for pervaporation process was compared to other desalination processes such as reverse osmosis (RO), multi stage flash (MSF) and multi effect distillation (MED) with the aim of identifying the potential applications/conditions that would be suitable for PV.

### 1.1. Energy Balance and Estimation

**Figure 1:**
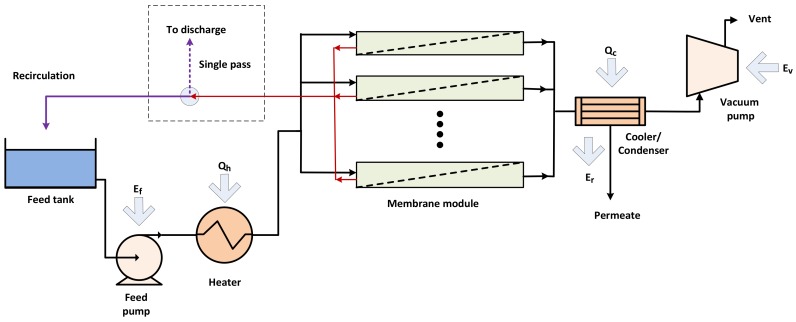
Schematic flow chart of pervaporation (PV) process in recirculation/single pass mode.

Figure 1 shows a schematic flow chart of the desalination by PV process in recirculation and single pass modes. The system consists of a feed pump, a heater, membrane modules, a cooler/condenser and a vacuum pump. In recirculation mode, the reject stream from the membrane modules is recirculated back to the feed tank, while in single pass mode the reject stream from the membrane modules is discharged and not recycled back to the process. The process engineering model considered only the major energy consuming components of the PV process that contribute to the desalination performance.

**Figure 2:**
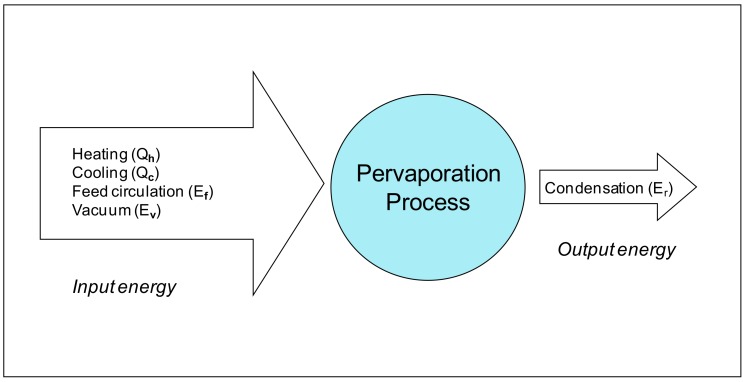
Breakdown of energy required for PV.

As shown in Figure 1 and Figure 2, major energy-consuming components for the PV process include heating of the feed stream, cooling/condensing of the permeate stream, the feed pump and vacuum pump. Feed heating (*Q_h_*) and permeate cooling energy (*Q_c_*) are classified as thermal energy requirements whereas the electrical power associated with feed pump (*E_f_*) and vacuum pump (*E_v_*) are classified as the electrical energy requirement. The latent heat of condensation (*E_r_*) from cooling the water vapor of the outlet permeate stream could be potentially recovered in the process and used for heating the feed.

The overall energy requirement is therefore:

(1) $$E_{Total\;}=Q_{h}+Q_{c}+E_{f}+E_{v}$$

If the heat recovery option is considered, depending on the heat recovery efficiency (*x*%), *E_Total_* could be calculated from:

(2) $$\;E_{Total\;}=Q_{h}+Q_{c}+E_{f}+E_{v}-x\%E_{r}$$

#### 1.1.1. Heating Energy Required for Heating the Feed Stream

In PV, permeate is in the vapor phase and the energy for this phase change is supplied by the sensible heat of the feed [10]. In addition, PV normally operates at a temperature higher than ambient temperature which is normally the temperature of the feed reservoir. In this case, energy is required to raise the temperature of the feed stream to the PV operating temperature (*T_fi_*). The energy required to heat the feed stream in the PV process can be calculated based on the operating conditions of the PV (feed flow rate and feed inlet/outlet temperatures). When the feed stream is recirculated (Figure 1), a one-off heating (*Q_init_*) is required to increase the temperature of the feed reservoir (*T_Res_*) to the desired operating feed temperature. At steady state, a heating energy (*Q_h_*) is required to compensate for the heat loss and boost the recirculating stream to the desired feed temperature:

(3) $$Q_{init\;}=m_{f}C_{pf}(T_{fi}-T_{Res})$$

(4) $$Q_{h}=m_{f}C_{pf\;}\left(T_{fi}-T_{fo}\right)$$

where *m_f_* is the mass flow rate of the feed (kg s^−1^), *C_pf_* is its heat capacity (J kg^−1^ K^−1^), *T_Res_ T_fi_* and *T_fo_* are the temperatures of the feed reservoir, feed inlet and feed outlet (K), respectively. Lower feed reservoir temperatures will increase the energy requirement for heating and vice versa.

#### 1.1.2. Cooling Energy Required for Permeate Condensation/Cooling

In PV, the water vapor on the permeate side needs to be condensed. Theoretically this portion of energy consumption equals the total energy used to evaporate permeate from the feed stream [13]. Condensation is normally carried out at temperature between 0 and 10 °C to achieve the desired operating permeate pressure. This condensing temperature is lower than that of cooling tower water. Therefore, further energy is required. However, this can often be minimized by using low temperature cooling devices which are often available on industry sites, such as in the fine chemical industry [10]. The thermal energy required to condensate the water vapor (*Q_c_*) is calculated as:

(5) $$Q_{c}=m_{p}\lambda \;$$

where *m_p_* is the mass flow rate of the permeate (kg s^−1^), and *λ* is latent heat of condensation of water vapor at the permeate temperature (J kg^−1^). When the temperature of condensed permeate stream is to be lowered, additional heat needs to be removed. The sensible heat released in the condenser comprises two parts: desuperheating from *T_pi_* to condensation temperature and subcooling from condensation temperature to *T_po_*, thus *Q_c_* becomes:

(6) $$Q_{c}=m_{p}\lambda +m_{p}\int_{T_{pi\;}}^{T_{pc}}C_{p,g}dT+m_{p}\int_{T_{pc}}^{T_{po}}C_{p,l}dT$$

where *T_pi_*, *T_pc_* and *T_po_* are the permeate inlet, condensation and out temperatures and *C_p_*_,*g*_ and *C_p_*_,*l*_ are the heat capacity of water vapor and liquid, respectively.

#### 1.1.3. Electrical Energy Required for Circulating the Feed Stream

The feed side pump is used to circulate the feed stream to the membrane module and overcome the pressure head loss across the membrane module. The electrical power consumption required for circulating the feed stream through the PV system is a function of the pressure drop and the volumetric flow rate of the feed. It was calculated as [9]:

(7) $$E_{f}=\frac{\Delta P_{f}V_{f}\;}{\eta_{p1}}$$

where *V_f_* is the volumetric flow rate of feed, *η_p_*_1_ is the pump efficiency which is assumed to be 80%, and *ΔP_f_* is the pressure drop in the pipe due to friction determined by [14]:

(8) $$\Delta P_{f}=f\frac{L}{D_{H}}\rho \frac{u^{2}}{2}$$

In the above equation, *f* is the Darcy’s friction factor, *L* is the channel length, *D_H_* is the hydraulic diameter, *ρ* is the density, and *u* is the linear velocity of the feed. For stream velocity in the laminar region (*Re* < 2100), the following correlation is applied:

(9) $$f=\frac{64\;}{Re}$$

with the *Re* defined as:

(10) $$Re=\frac{u\rho D_{H}\;}{\mu }$$

*μ* is the fluid viscosity and the hydraulic diameter, *D_H_*, is calculated from the geometry of the flow channel.

For turbulent flows (*Re* > 2100), the pressure drop is also affected by the changes in the feed channel such as the expansion, constriction, joint, and valves.

(11) ∑f=f(Re,εD)+∑(ev )

where the first term on the right hand side refers to the friction loss due to the material of the piping or tubing and can be estimated from the Moody chart based on the knowledge of the Reynolds number, in which ε is the surface roughness and *D* is the pipe diameter. For common polymeric materials, a smooth surface can be assumed. The second term on the right hand side of the equation (*e_v_*) represents the friction loss factor due to the disturbances in the flow channel. Some common values are listed in Table 2.

#### 1.1.4. Electrical Energy Required for Vacuum Pump

The electrical power consumption of a vacuum pump in the PV unit can be estimated based on the principle of adiabatic vapor expansion and contraction from the following equations [16]:

(12) $$E_{v}=-m_{nc\;}\int_{T_{in}}^{T_{out}}C_{p}dT$$

(13) $$T_{out\;}=T_{in}\left[1+\frac{1}{\eta_{p2}}\left({\left(\frac{p_{out}}{p_{in}}\right)}^{(\gamma -1)/\gamma }-1\right)\right]$$

where *m_nc_* is non-condensable flow rate (mol s^−1^), *C_p_* is the heat capacity of non-condensables at constant pressure (J mol^−1^ K^−1^), *T_in_* and *T_out_* are the vacuum pump inlet and outlet temperatures, *η_p_*_2_ is the vacuum pump efficiency which is assumed to be 80%, *p_out_* is the vacuum pump outlet pressure (normally atmospheric pressure), *p_in_* is the vacuum pump inlet pressure, and *γ* is the adiabatic expansion coefficient defined as [16]:

(14) $$\gamma =\frac{C_{p}\;}{C_{p}-R}$$

where *R* is the gas constant (8.3145 J mol^−1^K^−1^).

In PV, the vacuum pump is generally used after a condenser/cooler for start-up and removal of non-condensable vapors. For desalination applications, as the permeate is the water vapor which is condensable, the proportion of non-condensable vapor will be small. Non-condensable vapor mainly includes dissolved non-condensable gases from the feed stream and air leakage from the vacuum system. Consequently the power required for the vacuum pump will be very low at steady state as the condenser predominantly maintains the vacuum by efficient condensation of the permeate [8]. The dissolved non-condensable gases primarily include nitrogen and carbon dioxide (CO_2_) dissolved in the feed stream. The solubility of nitrogen and CO_2_ in water at the feed inlet temperature was used to estimate the dissolved non-condensable gases from the feed and air leaking into the vacuum system, expressed as *x*% mf. For example, at 20 °C, the solubility of air and CO_2_ in water are 0.023 g/kg and 1.72 g/kg, respectively. Gas solubility normally decreases with increasing temperature. At 60 °C, the solubility of air and CO_2_ in water drop down to 0.013 g/kg and 0.71 g/kg, respectively [17,18]. The above equations for calculating the electrical power consumption of the vacuum pump can then be simplified to the following equation [8,19]:

(15) Ev=x%mf R Tin ηp2AmMWγγ−1[(poutpin)(γ−1)γ−1]

where *J* is the permeate flux (kg/m^2^h), *T_in_* is the absolute temperature of the non-condensable vapor at the vacuum pump inlet condition (K) and *MW* is the molecular weight of the non-condensable gases (assumed to be air).

## 2. Materials and Methods

The hybrid PVA/MA/TEOS membrane used in this study contained 5 wt % MA and 10 wt % silica relative to PVA and was 20 μm thick. The synthesis method and conditions has been detailed elsewhere [20,21]. The synthesized membrane was heat treated 2 h at 140 °C prior to use. The PV test rig was described previously [20,21]. NaCl concentration of 0.2 wt % was used as the feed solution in all experiments. The large thickness of the membrane (20 μm) results in conservative estimates of flux and thinner supported membranes could achieve a higher flux. The feed flowrate was chosen such that the hydrodynamic conditions were fully developed and further increases in feed velocity resulted in no change in water flux.

## 3. Results and Discussion

### 3.1. Membrane Performance

**Figure 3:**
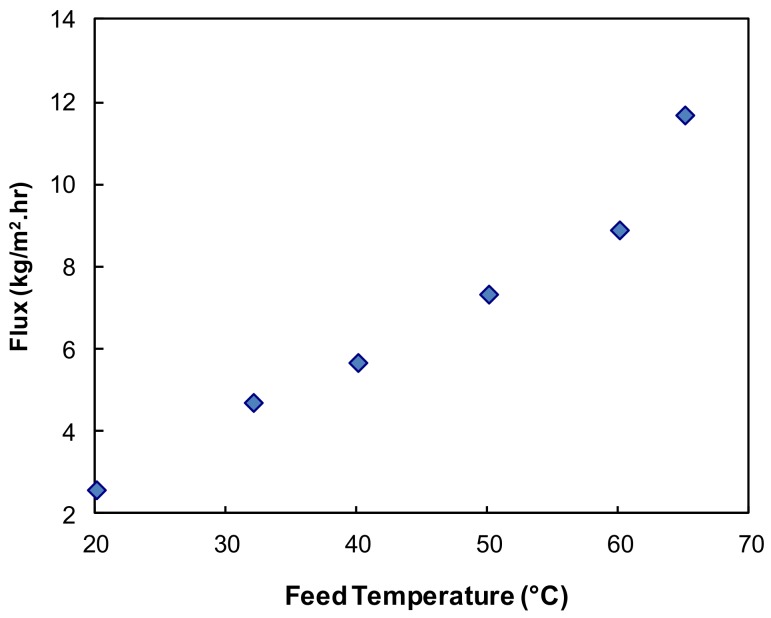
Effect of feed temperature on water flux (membrane thickness 20 µm, feed velocity 0.05 m/s, vacuum 6 Torr).

Figure 3 shows the effect of feed temperature on PV performance of hybrid PVA/MA/silica membrane at a feed velocity of 0.05 m/s and a vacuum 6 Torr. When the feed temperature was increased from 21 °C to 65 °C selected based on the laboratory room temperature and highest available industrial waste stream temperature, the water flux increased exponentially. A high water flux of 11.7 kg/m^2^·h was achieved at a feed temperature of 65 °C. The driving force for the pervaporation process is the partial vapor pressure difference of permeant between the feed and permeate conditions. As the feed temperature increased, the water vapor pressure on the feed side increased exponentially. As the vapor pressure on the permeate side was held constant, the increasing vapor pressure in feed led to an increase in the driving force and consequently the water flux. An increase in temperature also raises the diffusion coefficient for transport through the membrane, making it easier for the transport of the water molecules, therefore increasing the water flux by increasing the rate of mass transfer.

**Figure 4:**
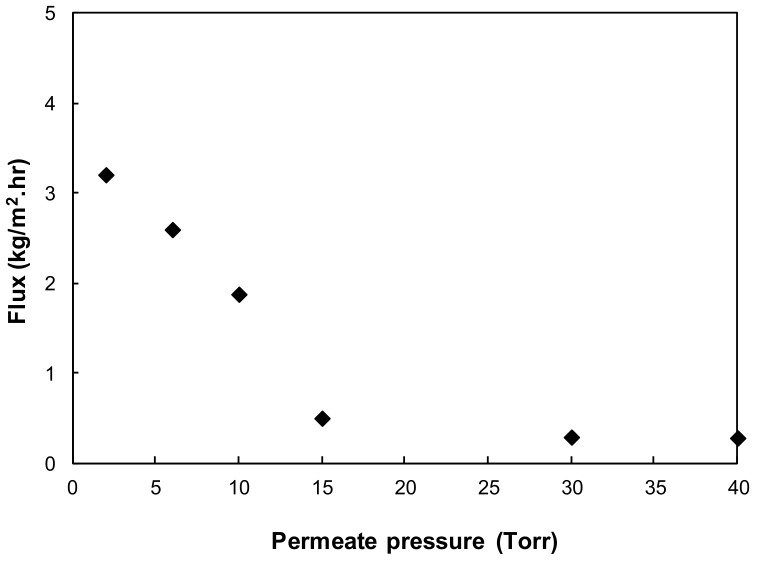
Effect of vacuum on water flux (membrane thickness 20 µm, feed temperature 21 °C, feed velocity 0.05 m/s).

Figure 4 shows the effect of permeate pressure on water flux. The water flux increased as the permeate pressure decreased. For PV, the driving force is provided by the vapor pressure difference between the feed and permeate side of the membrane. With decreasing permeate pressure (i.e., increasing vacuum), as the feed side vapor pressure remains unchanged, the transmembrane vapor pressure difference is increased. This leads to an increased driving force and consequently an increased water flux, especially when the permeate pressure is reduced to less than 15 Torr, which is below the saturation vapor pressure of the feed water (17 Torr at 21 °C). As explained in [22], the vapor pressure at the feed/membrane interface on the feed side became greater than the vacuum pressure at membrane interface on the permeate side when the permeate pressure was lower than the saturation vapor pressure. This leads to a dramatic increase in vapor flow occurring as the water boils, and consequently a higher flux results.

In this study, irrespective of variations in operating conditions, the salt (NaCl) rejection of the fabricated hybrid PVA/MA/TEOS membrane remained high (about 99.9%). This was mainly due to the non-volatile nature of NaCl compound and hydrophilic nature of hybrid PVA/MA/TEOS membrane. In PV separation of aqueous salt solution, water molecules preferentially diffuse and permeate into the membrane. In addition, as reported previous [20], the incorporation of silica nanoparticles in the polymer chain disrupted the polymer chain packing and reduced the fractional free volume by reducing both the size and concentration of free volume elements, and consequently led to high salt rejection.

Previous studies on desalination by PV mainly focused on zeolite and amorphous silica based membranes due to their molecular-sieving structure on the order of the kinetic diameter of the species to be separated (dp = 3–5 Å) [23,24,25]. Drobek reported a high water flux (~6 kg/m^2^·h) for a 3.5 wt % NaCl solution at 75 °C feed temperature for a MFI-ZSM-5 zeolite membrane. However, it was found that this zeolite membrane was severely affected by the salt concentration of the feed and temperature cycling, and displayed poor stability with low salt rejection (<90%) for desalination process. On the other hand, MFI-silicate-1 membrane exhibited high salt rejection (>99%) but low water flux (<5 kg/m^2^·h) [25]. Elma et al. [26] have conducted a comprehensive review on microporous silica based membranes for desalination via PV recently. Carbonized template silica membranes gave water fluxes varying from 1.4 to 6.3 kg/m^2^·h with high salt rejection greater than 84%, depending on the operating conditions [26,27,28]. The hybrid membrane prepared with BTESE showed very high water flux of 34 kg/m^2^·h at 90 °C but decreased to 3 kg/m^2^·h when cooler feed temperature (30 °C) was used [26,29]. CoO_x_Si based silica membranes gave a water flux from 1.8 to 0.55 kg/m^2^·h at 75 °C when the salt concentration ranged from 7.5 to 15% [26,30]. In general, the majority of silica–based membranes tested under PV desalination conditions did not produce pure water in the permeate stream [26]. Compared with these reported membranes, the high water flux (11.7 kg/m^2^·h at 65 °C feed temperature) and high salt rejection (99.9%) achieve by the PVA/MS/TEOS membranes in this study shows very promising results for its application in desalination by PV.

### 3.2. Specific Energy Requirement

**Figure 5:**
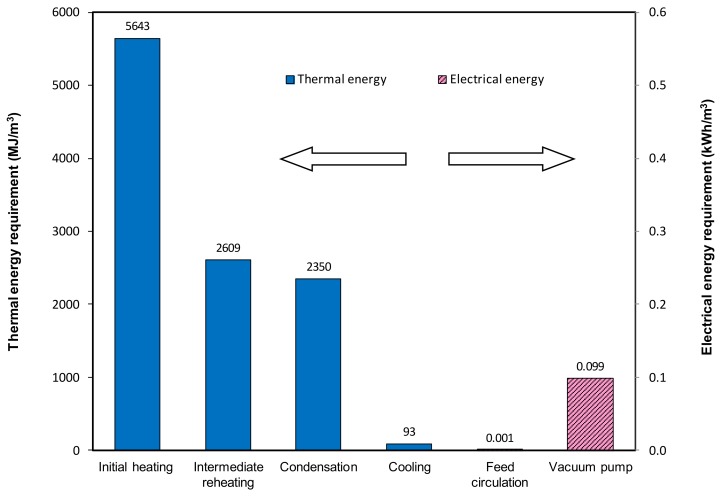
Breakdown of thermal and electrical energy requirement for PV process per m^3^ of permeate produced in recirculation mode (feed temperature 65 °C, feed velocity 0.05 m/s, vacuum 6 Torr).

The PV test results obtained on the PVA/MA/TEOS membrane in this study have been used as the basis to estimate the energy consumption required for desalination by PV. The energy required for PV is divided into thermal energy and electrical energy. The thermal energy includes initial heating of the feed stream from the feed reservoir temperature to the desired feed inlet temperature, intermediate re-heating of the feed stream to compensate for heat loss and to maintain the desired feed inlet temperature during recirculation, and the cooling energy required to condense and cool the permeate stream. The electrical energy includes the power associated with the feed and vacuum pumps. Figure 5 shows a breakdown of the thermal energy required and electrical power consumption for desalination by PV in recirculation mode using the experimental water flux of 11.7 kg/m^2^·h and an evaporation efficiency of 90% at a feed inlet temperature of 65 °C, feed velocity 0.05 m/s and vacuum level 6 Torr (800 Pa). The thermal energy required is significant. 5643 MJ/m^3^ is required to heat the feed reservoir from 21 °C to 65 °C initially and 2609 MJ/m^3^ of thermal energy is required to maintain the feed stream at 65 °C. It is noteworthy that the thermal energy values are indicative only and highly variable depending on the flux obtained experimentally. The heat of condensation removed in the condenser is almost equal to the intermediate heat required for permeate evaporation, with 2350 MJ/m^3^ required for permeate condensation and 93 MJ/m^3^ for cooling the permeate stream. In terms of electrical energy required, the vacuum pump requires most of the power and its value is about 0.099 kWh/m^3^. The circulation power required for the feed pump is negligible (<10% of total electrical energy requirement) compared with the vacuum pump energy.

**Figure 6:**
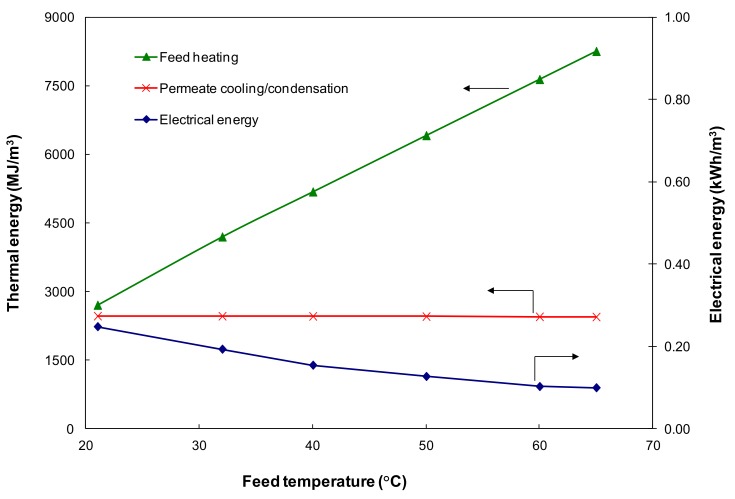
Effect of the feed temperature on thermal and electrical energy requirement (feed velocity 0.05 m/s, vacuum 6 Torr).

Figure 6 shows the effect of feed temperature on feed heating (including initial heating and intermediate reheating for the feed stream), permeate cooling/condensation thermal energy, and the electrical power required for operating the PV process at a feed velocity of 0.05 m/s and a 6 Torr permeate pressure. As the feed temperature increases from 21 to 65 °C at constant feed velocity and permeate pressure, the initial feed heating required increases from none to 5643 MJ/m^3^ of permeate to bring the feed from ambient temperature (21 °C) to 65 °C. The intermediate heating changed from 2701 to 2609 MJ/m^3^ of permeate as a result of the enthalpy change for the heat of vaporization. Combined together, more thermal heating energy is required with increasing feed temperature, from 2701 MJ/m^3^ at 21 °C to 8252 MJ/m^3^ at 65 °C. In addition, more heat is transferred from the feed stream, across the membrane and to the permeate stream resulting in an increase in permeate temperature. Therefore, more cooling energy is required to remove the heat from the permeate stream at higher feed temperature to the condensation temperature. When the feed temperature increases from 21 to 65 °C, sensible cooling energy increases from 19 to 93 MJ/m^3^. However, this increase is only marginal as the majority of the cooling energy (~2350 MJ/m^3^) is used for permeate condensation, which was relatively constant.

The feed temperature has less influence on the electrical energy requirement. When the feed inlet temperature increases from 21 to 65 °C, it was found that the power consumption reduced slightly for both the vacuum pump and the feed circulation pump, with the total electrical energy required decreasing from 0.25 to 0.10 kWh/m^3^. This is because the power consumption for the vacuum pump mainly depends on the pump inlet pressure and permeate flowrate (Equation (15)). The vacuum pump directly affects the permeate pressure and the pressure affects permeate flowrate. At a fixed production capacity (i.e., permeate flowrate) and permeate pressure (i.e., pump inlet pressure), the electrical power required by the vacuum pump per unit of product water remains unchanged (i.e., it is not a function of temperature). The main reason for the slight decrease is due to the solubility change of non-condensable gases dissolved in the feed stream. On the other hand, the electrical power required for the feed recirculation pump decreases with the increasing feed inlet temperature, from 0.002 at 21 °C to 0.001 kWh/m^3^ at 65 °C due to the viscosity reduced from 1.002 mPa·s to 0.4356 mPa·s and consequently increased *Re* at higher temperatures. However, this decrease is negligible as there in only a marginal increase of *Re* during the fully developed laminar flow region.

**Figure 7:**
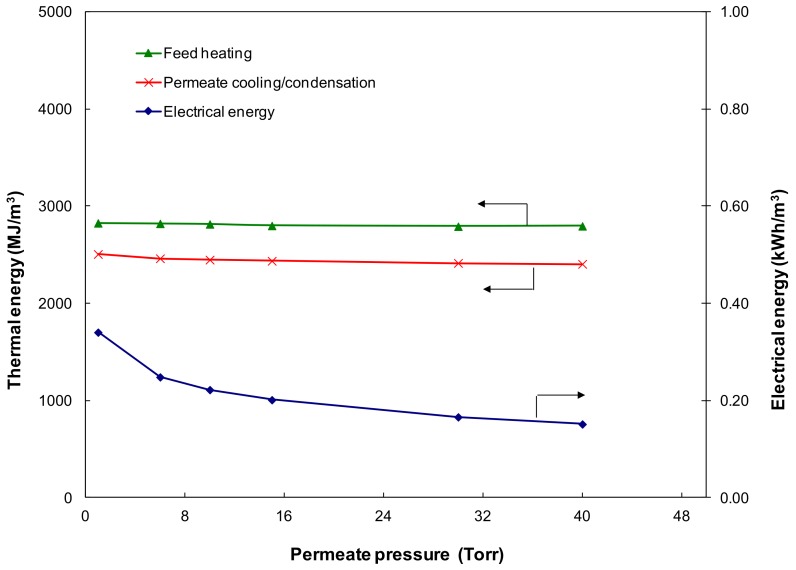
Effect of the permeate pressure on thermal and electrical energy requirement (feed velocity 0.05 m/s, feed temperature 21°C).

Figure 7 shows the effect of permeate pressure on feed heating, permeate cooling/condensation thermal energy, and the electrical power required for operating the pervaporation process at an ambient feed temperature (21 °C) and 0.05 m/s feed velocity. The thermal energy (both feed heating and permeate cooling) remains constant while the electrical energy required decreases continuously with increasing downstream permeate pressure. This is because, at a fixed production capacity, thermal energy is only related to the feed inlet temperature and permeate temperature which are normally constant.

The permeate pressure has negligible influence on the electrical power required for the feed recirculation pump due to the constant feed flowrate and pressure drop. As permeate pressure is increased from 1 to 40 Torr, the electrical power required for the vacuum pump decreased from 0.34 to 0.15 kWh/m^3^. As mentioned earlier, the power consumption for the vacuum pump is only related to the pump inlet pressure at a given production capacity. Higher permeate pressure indicates that less power is required to operate the vacuum pump. However, it should be noted that the water flux also decreases at higher permeate pressure due to the lower driving force, especially when the permeate pressure is below the saturation pressure; e.g., the water flux at room temperature dropped to ~0.29 kg/m^2^·h when the permeate pressure was more than the water saturation pressure of 17 Torr (Figure 4).

The thermal energy requirement is significant in PV processes as energy is required to increase the temperature of the feed water (and ultimately vaporize it), and also condense the water vapor. If the required thermal energy is supplied through conventional means, pervaporation would use more energy per kL of water produced than RO processes, which only use pump energy to pressurize the water feed and the cost will be prohibitive [26]. To reduce the process energy required, a portion or majority of this high-energy demand could be provided by (1) using low-grade or waste heat for heating the feed; (2) adopting a heat recovery option.

Table 3 compares the thermal and electrical energy with or without a heat recovery option and the use of a waste heat source. The heating energy required for the feed could be provided by energy sources such as waste heat from industrial sites and thermal power stations, salt gradient solar ponds or solar heat. Assuming low grade waste heat is readily available, the thermal energy is significantly reduced with only cooling energy required for permeate condensation. For example, this will reduce the total thermal energy from 5163 MJ/m^3^ to 2463 MJ/m^3^ of permeate at 21 °C (Table 3). It should be noted that the value of the low-grade thermal energy becomes higher as its temperature increases. It is, therefore, very important to improve the membrane performance as it lowers the required feed temperature and the specific membrane area. Consequently, this reduces the required low-grade thermal energy and the membrane related capital and operating cost.

In addition, if the heat recovery option is adopted to recover the latent heat of condensation gained in the condenser, the total energy required for the system could be potentially reduced down to a much improved level. That is, only the electrical power consumption for the vacuum pump and a small amount of thermal energy for cooling the permeate is required when ignoring any limitation of heat recovery from the permeate stream. For example, assuming 80% latent heat of permeate vapor could be recovered, only 526 MJ/m^3^ of thermal energy (cooling energy) and 0.25 kWh/m^3^ of electrical energy are required at 21 °C feed temperature and 6 Torr permeate pressure in a laminar flow regime (Table 3). However, it is worth noting that the heat recovery in pervaporation process is not straight forward as thermal desalination technologies such as MED or MSF. In PV, the permeate temperature is generally lower than the feed temperature and the latent heat of condensation cannot be directly reused for heating the feed; e.g., at the vacuum pressure of 6 Torr used in this study—the condensation temperature of water is only 4 °C, which is lower than the temperature of the fresh feed from the ambient (21 °C) and the temperature of the recirculating stream from the membrane module. In this case, the heat recovery technologies such as heat pump could be used to increase the temperature of a waste-heat stream to a higher, more useful temperature and to recover latent heat from high-humidity streams [31]. However, the effective heat recovery could be a lot lower as the additional energy would be needed to drive the heat pump.

Table 4 compares the energy consumption of PV against other desalination technologies such as RO, MSF, MED and VC. Except for RO using electrical power only, the energy used in other desalination processes such as MSF, MED, and VC consist of thermal and electrical energy [32]. As can be seen, the energy needed for RO is considerably lower than the distillation options. However, its electrical energy requirement is still significant. Survey data of desalination plants operating in Australia has shown that the average energy consumption was 2.2 kWh/kL for seawater RO, 0.7–1 kWh/kL for brackish water and 1.2 kWh/kL for industrial effluents [4]. The electrical energy needed for PV is the lowest among these desalination technologies (<0.3 kWh/kL). Kaminski et.al [33] recently did a comparison of energy consumption on desalination via PV, RO, and MD. They also found that PV technology prevailed for its low electrical energy consumptions, but the thermal energy requirement between the technologies was not directly comparable as they could be incredibly vast depending on individual process conditions. Based on our study, without a heat recovery option, the thermal energy needed for pervaporation is the highest despite its low electrical energy requirement. With the option of a free low grade waste heat source and potential heat recovery option, the PV could become economical with the thermal energy being comparable to thermal distillation technologies but with much lower electrical energy.

These results indicate PV cannot compete with RO directly in terms of energy consumption when utilizing conventional energy sources especially for use in brackish/seawater desalination where NaCl concentration is below 6 g/L, due to the considerable latent heat (2618 kJ/kg at 65 °C) needed for evaporation. However, when PV processes are integrated with waste heat or solar heat sources and heat recovery options are adopted (e.g., using a Thermo PV process where heat energy could be recovered up to 33% (in ethanol separation)) [34], the technology may be attractive, especially for high salinity feeds where RO energy requirements increase while PV energy requirements are essentially independent of the salt concentration in the feed solution. This suggests that pervaporation could be applied in niche markets where RO has limitations, such as RO brine concentration, salt recovery, or the area requiring zero brine discharge. For instance, it has been proven that PV desalination is feasible to treat produced water from mineral oil and natural gas extraction when salinity is as high as 400 g/L, which makes it difficult for RO process because of the requirement of high hydraulic pressure to overcome osmotic pressure [35].

Hybrid system incorporating PV with conventional thermal processes is another way of benefiting from the membrane system. Simulation studies have shown that these hybrid systems can be considered as a real alternative to cheaper and environmentally friendly processes. For instance, a distillation and PV hybrid system was able to save up to 86% of the total energy requirement compared to the conventional pressure swing distillation process [36]. Recent modelling study by Felicia N. et al. has proven that their hybrid extractive distillation column with a PV system could save up to 25% and 41% of total annual cost and energy, respectively, in the alcohol dehydration process [37]. Another niche application is to recover alcohol straight from the its fermentation broth [38,39]. Researchers have discovered that an in-situ incorporation of the PV membrane system to the fermentation reactor can reduce energy costs whilst attaining efficient alcohol solvent recovery. Having said all that, much improvement is still needed in terms of innovative membrane material discovery, smart modules design, and efficiently engineered processes, before PV is ready to take on the currently available best desalination technologies.

### 3.3. Single Pass versus Recirculation

**Figure 8:**
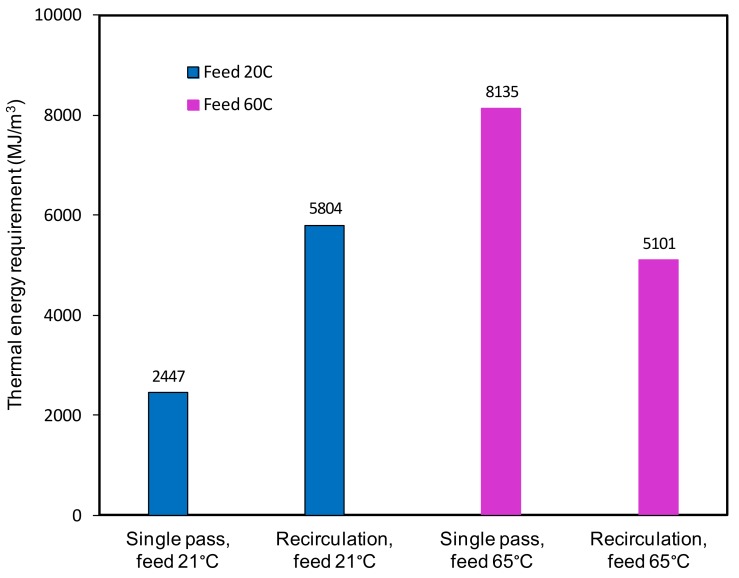
Thermal energy requirement per m^3^ permeate for single pass and recirculation mode (feed velocity 0.05 m/s, vacuum 6 Torr).

Whether to operate the system in single pass or recirculation mode needs also to be considered during process design. The electrical energy required will remain relatively constant regardless of the mode of operation as the flows associated with the electrical power consumption remains stable. However, the mode of operation will have an impact on the thermal energy required, which varies with the feed temperature. In single pass mode, the reject stream from the membrane module is discharged. On the other hand, this stream is recirculated back to the feed reservoir in recirculation mode at a higher temperature than the fresh feed. Therefore, while only initial heat is required to bring the feed stream to the required feed temperature in single pass, extra thermal energy is required to compensate the heat loss of the feed stream in recirculation mode. Figure 8 compares the thermal energy required for single pass and recirculation mode at two different feed inlet temperatures (21 °C and 65 °C) based on lab scale experiment results in the absence of waste heat and any heat recovery. At low feed temperature (<30 °C), negligible or minimum amount of heating is required for the feed stream in single pass. However, greater heating is required in recirculation mode as the recirculated feed is returned at a lower temperature than the fresh feed and therefore requires additional heating to compensate the heat loss. On the other hand, the opposite is true at a higher feed temperature (65 °C), more thermal energy is required in the single pass mode than the recirculation mode. This is because, at higher temperatures, the recirculated feed is returned at a temperature higher than the fresh feed and therefore requires less heat. On the other hand, the feed stream always needs to be heated from an ambient temperature to the desired feed inlet temperature in a single pass, resulting in greater required energy. In addition, lower water recoveries and large quantities of concentrate also need to be discharged for single pass operation. Thus, the recirculation mode is the preferred configuration at higher feed temperatures. Moreover, the recirculation mode is generally preferred regardless of the feed temperature as discharging large quantities of the reject stream in a single pass can be avoided in recirculation mode. It is worth noting the recirculation temperature is based on current laboratory study and the actual temperature of the concentrate stream before circulation will vary with the module size and consequently the actual energy requirement.

## 4. Conclusions

A process engineering model was developed to assess the specific energy required for desalination by PV which includes thermal and electrical energy. The thermal energy required is significant, e.g., in recirculation mode, a significant initial thermal energy is required to heat the feed reservoir from 21 °C to 65 °C to start up the process and 2609 MJ/m^3^ is required to maintain the feed stream at 65 °C. At steady state, the electrical energy requirement is very small. In the studied laminar flow regime, the vacuum pump contributes to the majority of the electrical energy, with 0.1 kWh/m^3^ of electrical power required at 65 °C feed temperature. To reduce the process energy requirement, low grade waste heat sources and heat recovery could be used to provide the thermal heating and recover the heat of condensation in the condenser. With the option of a free waste heat source and potential heat recovery, the thermal energy needed for PV could be comparable to other thermal desalination technologies but with minimal electrical energy consumption. It was found that that pervaporation cannot directly compete with RO technology without a free waste heat resource. However, PV could be potentially applied in niche markets where RO has limitations, such as RO brine concentration, salt recovery or the applications requiring zero brine discharge.

Operating conditions such as feed inlet temperature and permeate pressure have different effects on the specific energy required for the PV process. Thermal energy increases with increasing feed temperature but remains constant with changing permeate pressure. On the other hand, the electrical energy decreases with both increasing temperature and permeate pressure. In scale up operation, the recirculation mode is generally preferred as it has the advantage of reducing the discharge of large quantities of the brine and reducing the thermal energy requirement when high temperature waste heat is available. Operating the system in single pass mode will only have an advantage at low feed temperatures where the initial heating is not required and a waste heat resource is not available.

## Figures and Tables

**Table 1:** Summary of desalination technologies [3].

Parameter	Seawater RO	Brackish RO	MED	EDR
Feed water salinity (mg/L TDS)	>32,000	<32,000	>35,000	3000–12,000
Product water salinity (mg/L TDS)	<500	<200	<10	<10
Min. product water volume	500 L/day	500 L/day	120 kL/day	90 kL/day
% recovery	≤50	≥80	40–65	>90
Energy required	Electrical energy	Electrical energy	Thermal energy or waste heat energy	Electrical energy
Typical capital Cost (A$/kL/day of product water)	1600–2500	600–1800	2500–3900	570–3250
Typical operating cost (A$/kL of product water)	1.89–2.20	0.65–1.50	Without waste heat: 1.8–2.80 With waste heat: 0.55–0.95	1.00–2.80

**Table 2:** Common values for friction loss factors [15].

Disturbances	*e_v_*
Changes in cross-section area:	
Sudden contraction	0.45 (1–*β*) *
Sudden expansion	${\left(\frac{1}{\beta }-1\right)}^{2}$
Fittings and valves:	
90° round elbows	0.4–0.9
45° elbows	0.3–0.4
Open globe valve	6–10

* $\beta =\frac{\mathrm{smaller}\;\mathrm{cross}\;\mathrm{section}}{\mathrm{larger}\;\mathrm{cross}\;\mathrm{section}}$.

**Table 3:** Thermal and electrical energy requirement per m^3^ of permeate with/without heat recovery and alternative heat source.

Feed Temperature (°C)	Heating Energy (MJ/m^3^)		Cooling Energy (MJ/m^3^)		Electrical Energy (kWh/m^3^)
	No Waste Heat	With Waste Heat	No Heat Recovery	80% Heat Recovery	
21	2701	0	2462	526	0.25
32	4194	0	2460	562	0.19
40	5179	0	2457	586	0.15
50	6410	0	2452	614	0.13
60	7638	0	2447	644	0.10
65	8252	0	2443	656	0.10

**Table 4:** Energy consumption for various desalination technologies.

Process	Thermal ^1^ (MJ/m^3^)	Electrical ^1^ (kWh/m^3^)
Multi stage flash (MSF)	250–300	3.5–5
Multi effect distillation (MED)	150–220	1.5–2.5
Vapor compression (VC)–thermal	220–240	1.5–2
Vapor compression (VC)—mechanical	None	11–12
RO—seawater	None	2.2–3.7
RO—brackish water	None	0.7–1.2
PV		
waste heat, no heat recovery	2443–2462	0.1–0.25
waste heat, 80% heat recovery	526–656	0.1–0.25

^1^ Thermal and electrical energy of MSF, MED, VC and RO are extracted from [32].

## Nomenclature

A_m_	active membrane area (m^2^)
*C_p_*	non-condensable heat capacity (J/mol/K)
*C_pf_*	feed stream heat capacity (J/kg/K)
*C_p,g_*	water (vapour) heat capacity (J/kg/K)
*C_p,l_*	water (liquid) heat capacity (J/kg/K)
D	pipe diameter (m)
D_H_	hydraulic diameter (m)
*e_v_*	friction loss factor (dimensionless)
*E_f_*	feed pump electrical energy (J)
*E_r_*	latent heat of condensation (J)
*E_Total_*	overall energy requirement (J)
*E_v_*	vacuum pump electrical energy (J)
*f*	Darcy’s friction factor (dimensionless)
*J*	permeate flux (kg/m^2^·h)
*L*	channel length (m)
*m_f_*	mass flow rate of feed stream (kg/s)
*m_nc_*	non-condensable flow rate (mol/s)
*m_p_*	mass flow rate of permeate (kg/s)
*MW*	molecular weight of non-condensable gases (g/mol)
*P_in_*	vacuum pump inlet pressure (Pa)
*P_out_*	vacuum pump outlet pressure (Pa)
*Q_c_*	permeate stream cooling energy (J)
*Q_h_*	feed stream heating energy (J)
*Q*_init_	one-off initial heating energy (J)
*R*	universal gas constant (J/mol/K)
*Re*	Reynold’s number (dimensionless)
*T_fi_*	temperature of feed inlet (K)
*T_fo_*	temperature of feed outlet (K)
*T_in_*	temperature of vacuum pump inlet stream (K)
*T_pc_*	condensation temperature of permeate (K)
*T_pi_*	temperature of permeate inlet (K)
*T_po_*	temperature of permeate outlet (K)
*T_Res_*	temperature of feed reservoir (K)
*T_out_*	temperature of vacuum pump outlet stream (K)
*u*	linear velocity of feed stream (m/s)
*V_f_*	volumetric flow rate of feed stream (m^3^/s)
*ΔP_f_*	pressure drop of pipe due to friction (Pa)
ε	surface roughness (m)
*β*	smaller cross section/larger cross section (dimensionless)
γ	adiabatic expansion coefficient (dimensionless)
λ	latent heat of condensation of water vapour (J/kg)
*ρ*	fluid density (kg/m^3^)
μ	fluid viscosity (Pa.s)
*η*_*p*1_	feed pump efficiency (%)
*η*_*p*2_	vacuum pump efficiency (%)
*Subscripts*	
*fi*	feed inlet
*fo*	feed outlet
*pi*	permeate inlet
*po*	permeate outlet

## Abbreviations

BTESE	1,2-bis (triethoxysilyl)ethane
CDI	capacitive deionization
EDR	electrodialysis reversal
FO	forward osmosis
MA	maleic acid
MD	membrane distillation
MED	multiple effect distillation
MSF	multi-stage flash
NaCl	sodium chloride
PVA	polyvinyl alcohol
RO	reverse osmosis
TDS	total dissolve solid
TEOS	tetraethyl orthosilicate
VC	vapor compression

## References

[1] McGinnis R.L., Elimelech M. (2007). Energy requirements of ammonia-carbon dioxide forward osmosis desalination. Desalination.

[2] Karagiannis I.C., Soldatos P.G. (2008). Water desalination cost literature: Review and assessment. Desalination.

[3] Korngold E., Korin E., Ladizhensky I. (1996). Water desalination by pervaporation with hollow fiber membranes. Desalination.

[4] Hoang M., Bolto B., Haskard C., Barron O., Gray S., Leslie G. (2009). Desalination in Australia.

[5] Elimelech M., Phillip W.A. (2011). The Future of Seawater Desalination: Energy, Technology, and the Environment. Science.

[6] Fiorenza G., Sharma V.K., Braccio G. (2003). Techno-economic evaluation of a solar powered water desalination plant. Energy Convers. Manag..

[7] Suggala S.V., Bhattacharya P.K. (2003). Real coded genetic algorithm for optimization of pervaporation process parameters for removal of volatile organics from water. Ind. Eng. Chem. Res..

[8] Ji W.C., Hilaly A., Sikdar S.K., Hwang S.T. (1994). Optimization of Multicomponent Pervaporation for Removal of Volatile, Organic-Compounds from Water. J. Membr. Sci..

[9] Alklaibi A.M. (2008). The potential of membrane distillation as a stand-alone desalination process. Desalination.

[10] Howell J.A., Watt Committee on Energy, Working Group on Membranes (1990). The Membrane Alternative: Energy Implications for Industry.

[11] Criscuoli A., Carnevale M.C., Drioli E. (2008). Evaluation of energy requirements in membrane distillation. Chem. Eng. Process.

[12] Servel C., Roizard D., Favre E., Horbez D. (2014). Improved Energy Efficiency of a Hybrid Pervaporation/Distillation Process for Acetic Acid Production: Identification of Target Membrane Performances by Simulation. Ind. Eng. Chem. Res..

[13] Shao P., Kumar A. (2011). Process energy efficiency in pervaporative and vacuum membrane distillation separation of 2,3-butanediol. Can. J. Chem. Eng..

[14] Munson B.R., Young D.F., Okiishi T.H. (2002). Fundamentals of Fluid Mechanics.

[15] Bird R.B., Stewart W.E., Lightfoot E.N. (2007). Transport Phenomena.

[16] Chodkiatsakul I., Charojrochkul S., Kiatkittipong W., Wiyaratn W., Soottitantawat A., Arpornwichanop A., Laosiripojana N., Assabumrungrat S. (2011). Performance improvement of bioethanol-fuelled solid oxide fuel cell system by using penraporation. Int. J. Hydrogen Energy.

[17] Battino R., Rettich T.R., Tominaga T. (1984). The Solubility of Nitrogen and Air in Liquids. J. Phys. Chem. Ref. Data.

[18] Carroll J.J., Slupsky J.D., Mather A.E. (1991). The Solubility of Carbon-Dioxide in Water at Low-Pressure. J. Phys. Chem. Ref. Data.

[19] Vallieres C., Favre E. (2004). Vacuum versus sweeping gas operation for binary mixtures separation by dense membrane processes. J. Membr. Sci..

[20] Xie Z.L., Hoang M., Duong T., Ng D., Dao B., Gray S. (2011). Sol-gel derived poly(vinyl alcohol)/maleic acid/silica hybrid membrane for desalination by pervaporation. J. Membr. Sci..

[21] Xie Z.L., Ng D., Hoang M., Duong T., Gray S. (2011). Separation of aqueous salt solution by pervaporation through hybrid organic-inorganic membrane: Effect of operating conditions. Desalination.

[22] Zhang J.H., Li J.D., Duke M., Hoang M., Xie Z.L., Groth A., Tun C., Gray S. (2013). Modelling of vacuum membrane distillation. J. Membr. Sci..

[23] Lin J., Murad S. (2001). A computer simulation study of the separation of aqueous solutions using thin zeolite membranes. Mol. Phys..

[24] Duke M.C., Mee S., da Costa J.C.D. (2007). Performance of porous inorganic membranes in non-osmotic desalination. Water Res..

[25] Drobek M., Yacou C., Motuzas J., Julbe A., Ding L.P., da Costa J.C.D. (2012). Long term pervaporation desalination of tubular MFI zeolite membranes. J. Membr. Sci..

[26] Elma M., Yacou C., Wang D.K., Smart S., da Costa J.C.D. (2012). Microporous Silica Based Membranes for Desalination. Water.

[27] Wijaya S., Duke M.C., da Costa J.C.D. (2009). Carbonised template silica membranes for desalination. Desalination.

[28] Ladewig B.P., Tan Y.H., Lin C.X.C., Ladewig K., da Costa J.C.D., Smart S. (2011). Preparation, Characterization and Performance of Templated Silica Membranes in Non-Osmotic Desalination. Materials.

[29] Lin C.X.C., Ding L.P., Smart S., da Costa J.C.D. (2012). Cobalt oxide silica membranes for desalination. J. Colloid Interface Sci..

[30] Tsuru T., Igi R., Kanezashi M., Yoshioka T., Fujisaki S., Iwamoto Y. (2011). Permeation Properties of Hydrogen and Water Vapor Through Porous Silica Membranes at High Temperatures. AIChE J..

[31] Silva A.M., Rosa R. (1993). Heat-Pumps for Efficient Energy Use in Industrial-Processes and for Process Integration. Heat Pumps for Energy Efficiency and Environmental Progress.

[32] Khalifa A.J.N. (2011). Evaluation of different hybrid power scenarios to Reverse Osmosis (RO) desalination units in isolated areas in Iraq. Energy Sustain. Dev..

[33] Kaminski W., Marszalek J., Tomczak E. (2018). Water desalination by pervaporation—Comparison of energy consumption. Desalination.

[34] Fernandez E.S., Geerdink P., Goetheer E.L.V. (2010). Thermo pervap: The next step in energy efficient pervaporation. Desalination.

[35] Gude G. (2018). Emerging Technologies for Sustainable Desalination Handbook.

[36] Luis P., Amelio A., Vreysen S., Calabro V., Van der Bruggen B. (2014). Simulation and environmental evaluation of process design: Distillation vs. hybrid distillation-pervaporation for methanol/tetrahydrofuran separation. Appl. Energy.

[37] Novita F.J., Lee H.-Y., Lee M. (2018). Energy-efficient and ecologically friendly hybrid extractive distillation using a pervaporation system for azeotropic feed compositions in alcohol dehydration process. J. Taiwan Inst. Chem. Eng..

[38] Cai D., Hu S., Miao Q., Chen C.J., Chen H.D., Zhang C.W., Li P., Qin P.Y., Tan T.W. (2017). Two-stage pervaporation process for effective in situ removal acetone-butanol-ethanol from fermentation broth. Bioresour. Technol..

[39] Fan S.Q., Xiao Z.Y., Li M.H., Li S.Z. (2017). Ethanol fermentation coupled with pervaporation by energy efficient mechanical vapor compression. Energy Proc..

